# 初治非手术小细胞肺癌复发/进展影响因素的回顾性分析

**DOI:** 10.3779/j.issn.1009-3419.2015.09.01

**Published:** 2015-09-20

**Authors:** 亚琴 庄, 丽岩 姜, 怡卓 赵, 嘉琳 钱, 习文 高

**Affiliations:** 1 200030 上海，上海交通大学附属胸科医院肺内科 Department of Pulmonary Medicine, Shanghai Chest Hospital, Shanghai Jiao Tong University, Shanghai 200030, China; 2 201199 上海，上海市闵行区中心医院呼吸内科 Department of Respiratory, Central Hospital of Shanghai Minhang District, Shanghai 201199, China

**Keywords:** 肺肿瘤, 化疗, 放疗, 复发, Lung neoplasms, Chemotherapy, Radiotherapy, Relapse

## Abstract

**背景与目的:**

小细胞肺癌对初始放化疗敏感，但容易复发或转移，生存率低。本研究目的在于探讨初治非手术小细胞肺癌复发/进展的影响因素，并分析复发/进展时间（无进展生存时间）（progression-free survival, PFS）与总生存时间（overall survival, OS）之间的相关性。

**方法:**

回顾性分析182例于2009年1月-2011年12月在上海市胸科医院新诊断的住院并接受化疗联合/不联合放疗后出现复发/进展的非手术小细胞肺癌患者的临床资料，进行单因素和*Cox*回归多因素分析各种因素对PFS的影响。采用双变量相关分析来分析PFS与OS之间的关系。

**结果:**

单因素分析显示发病时肿瘤TNM分期、是否肝转移、是否脑转移、一线化疗周期数、初始化疗疗效以及是否胸腔放疗对PFS有影响。发病时非脑转移患者中接受预防性颅脑照射（prophylactic cranial irradiation, PCI）与未接受PCI对PFS存在统计学差异。多因素分析表明一线化疗周期数、初始化疗疗效、胸腔放疗与否是影响PFS的独立因素。双变量相关分析显示PFS与OS存在显著正相关。

**结论:**

一线化疗周期数多（大于4次）、初始化疗疗效好（部分缓解或完全缓解）、联合胸腔放疗以及非脑转移者行PCI可延长小细胞肺癌患者的PFS。

## Abstract (en)

**Background and objective:**

Most small cell lung cancer (SCLC) patients relapse or progress and have low survival rate although they have significant response to initial chemotherapy and radiotherapy. This study intends to explore the factors affecting the relapse (or progression) of nonoperative SCLC and to explore the correlations between progression-free survival (PFS) and overall survival (OS).

**Methods:**

Clinical data of 182 patients diagnosed with SCLC between January 2009 and December 2011 at Shanghai Chest Hospital has been reviewed and retrospectively analyzed. All of these patients accepted chemotherapy combined (or not combined) with radiotherapy, and relapsed or progressed after first-line therapy. Univariate *Kaplan-Meier* survival estimates as well as multivariate *Cox* regression survival analysis were used to locate the potential factors affecting PFS. The correlation between PFS and OS was analyzed via *Bivariate Correlation* Analysis method.

**Results:**

The univariate estimates showed that the TNM stage, liver metastasis or not, brain metastasis or not, first-line chemotherapy cycles, effect of initial chemotherapy, and thoracic radiotherapy combined or not were the significant contributive factors to PFS. In the subgroup of the patients without brain metastases, those received prophylactic cranial irradiation (PCI) had longer PFS. *Cox* regression indicated that the three independent variables of first-line chemotherapy cycles, effect of initial chemotherapy and thoracic radiotherapy combined or not were closely related to PFS. In addition, significant positive correlation between PFS and OS had been observed.

**Conclusion:**

PFS could be prolonged by having more first-line chemotherapy cycles (> 4 cycles), obtaining better effect of initial chemotherapy (partial response or complete response), combining with thoracic radiotherapy and implementing PCI for patients without brain metastasis.

小细胞肺癌（small cell lung cancer, SCLC）占肺癌的10%-15%，由于其进展快、恶性程度高、易早期远处转移，几乎2/3的患者发现时已处于广泛期，预后差。局限期SCLC的中位生存时间为15个月-20个月，5年生存率为15%或以下。广泛期SCLC的中位生存时间为9.4个月-12.8个月，2年生存率为5.2%-19.5%，仍旧是令人失望的^[1]^。化疗是该病的主要治疗基础，虽然起初对化疗和放疗相当敏感，SCLC在疾病进程中容易产生耐药，大部分患者在2年内复发并因全身转移而死亡。出现复发/进展后是否导致生存期缩短尚缺少相关文献报道。寻找SCLC复发/进展的高危因素以及影响SCLC复发/进展时间长短的治疗因素，从而更有效地评估每一位新诊断的SCLC患者，进行更具个体化的治疗，尽可能延长初治SCLC患者的无疾病进展时间，提高生存率，具有显著的临床意义。

本研究回顾性分析进行一线治疗（化疗联合/不联合放疗，并排除手术治疗）后出现复发/进展SCLC患者的临床资料，探讨SCLC复发/进展的影响因素，并分析复发/进展时间与总生存时间是否存在相关性。

## 1 资料与方法

### 1.1 临床资料

查阅在上海市胸科医院住院治疗并于2009年1月-2011年12月经组织病理学或细胞学新确诊的复发/进展SCLC患者的临床资料，共182例。男性156例，女性26例，中位年龄58.47岁。入选标准：①经组织病理学或细胞学检查确诊为SCLC；②必须接受过一线化疗；③为经化疗（伴/不伴放疗）后出现复发/进展患者；④排除外科手术患者（包括诊断性手术、术前明确为其他类型肺癌而行手术但术后病理提示混合有小细胞成分、SCLC直接手术者、SCLC化疗伴/不伴放疗后进一步手术者）；⑤排除不规律化疗者；⑥排除仅在胸科医院行放疗，外院化疗信息不详者；⑦排除主要观察指标数据不完整者。

### 1.2 研究方法

收集患者完整的临床资料，包括：①患者的一般特征：年龄、性别、吸烟情况、美国东部肿瘤协作组（Eastern Cooperative Oncology Group, ECOG）体能状态（performance status, PS）评分；②患者的肿瘤及生化相关信息：病理获取时间、病理分型（单纯型、复合型）、发病时肿瘤分期（TNM）、转移部位、转移器官数、发病时各指标水平[癌胚抗原（carcino-embryonic antigen, CEA）： > 5 ng/mL为升高；神经元特异性烯醇化酶（neuron specific enolase, NSE）： > 25 ng/mL为升高；乳酸脱氢酶（lactate dehydrogenase, LDH）： > 245 u/L为升高；白蛋白： < 34 g/L为降低；血红蛋白：女性 < 110 g/L，男性 < 120 g/L为降低；血钠水平： < 135 mmol/L为降低]；③患者的治疗相关信息：一线化疗方案（分为两组：两药组及三药组；两药组又分为卡铂组与顺铂组）、一线化疗周期数（分为两组：≤4及 > 4）、初始化疗疗效[评估标准：完全缓解（complete response, CR）；部分缓解（partial response, PR）；稳定（stable disease, SD）；进展（progressive disease, PD）]（分三组：CR+PR、SD、PD）、是否胸腔放疗、胸腔放疗介入时间（早期介入指≤化疗4个周期时放疗，晚期介入 > 化疗4个周期时放疗）、胸腔放化疗方式（同步放化疗、序贯放化疗）、肿瘤复发/进展时间（无进展生存时间）（progression-free survival, PFS）（指从病理获取时间至疾病进展或患者死亡的时间）、是否预防性颅脑照射（prophylactic cranial irradiation, PCI）、复发/进展部位、总生存时间（overall survival, OS）（指从病理获取时间至患者死亡或末次随访时间）；④采用电话随访形式，末次随访时间为2015年5月31日。

### 1.3 统计学方法

采用SPSS 22.0软件进行数据统计分析。采用单因素（*Kaplan-Meier*分析）以及*Cox*回归多因素分析各种因素对PFS及OS的影响。采用双变量相关分析SCLC PFS与OS之间的相关性。采用双变量相关分析来分析非脑转移患者是否行PCI与之后脑转移发生率之间的关系。以*P* < 0.05为差异有统计学意义。

## 2 结果

### 2.1 患者一般情况

符合入选标准患者共182例，全部接受化疗，其中78例接受胸腔放疗，23例接受PCI。患者年龄最小30岁，最大79岁，中位年龄58.47岁，PFS为1个月-60个月，中位时间为9.57个月；OS为4个月-84个月，中位时间为17.95个月。1年、2年、3年的生存率分别为60.44%、29.12%、17.58%。

#### 2.2.1 SCLC患者PFS与OS间的双变量相关分析

SCLC PFS与OS的*Pearson*相关系数为0.655（*P* < 0.001），两者间存在显著正相关。

#### 2.2.2 单因素分析

单因素分析显示发病时肿瘤TNM分期、肝转移与否、脑转移与否、一线化疗周期数、初始化疗疗效、是否胸腔放疗对PFS有影响；发病时血红蛋白水平、肿瘤TNM分期、肝转移与否、肺内转移与否、一线化疗周期数、初始化疗疗效、是否胸腔放疗对OS有影响（表 1）。把一线化疗方案为两药组患者进一步分成卡铂组与顺铂组后比较PFS及OS情况显示无统计学差异（表 2）。胸腔放疗介入时间及放化疗方式对PFS及OS无统计学差异（表 3）。

**1:** SCLC患者PFS及OS影响因素的单因素分析 Univariate analysis of PFS and OS in SCLC patients

Factor	*n*	PFS (95%Cl) (month)	*χ*^2^	*P*	OS（95%Cl）(month)	*χ*^2^	*P*
Gender			0.128	0.720		0.626	0.429
Male	156	9.44 (8.18-10.71)			17.51 (15.32-19.71)		
Female	26	10.35 (6.02-14.67)			20.58 (14.35-26.80)		
Age (yr)			1.243	0.265		0.907	0.341
≥60	85	8.95 (7.23-10.68)			16.85 (13.91-19.79)		
< 60	97	10.11 (8.34-11.89)			18.92 (15.99-21.84)		
Smoking index			1.725	0.189		0.286	0.593
≥400	117	8.79 (7.68-9.89)			17.52 (14.97-20.07)		
< 400	65	10.99 (8.15-13.82)			18.72 (15.14-22.31)		
ECOG PS			0.243	0.622		1.580	0.209
0-1	180	9.59 (8.34-10.85)			18.04 (15.95-20.14)		
≥2	2	7.50 (4.56-10.44)			9.50 (4.60-14.40)		
LDH			3.435	0.064		0.705	0.401
High	48	8.00 (6.29-9.71)			16.73 (12.91-20.55)		
Normal	134	10.13 (8.57-11.70)			18.39 (15.92-20.86)		
Hyponatremia			0.291	0.590		1.015	0.314
Yes	20	12.10 (4.73-19.47)			22.25 (13.80-30.69)		
No	162	9.26 (8.19-10.33)			17.42 (15.33-19.51)		
Hemoglobin			1.023	0.312		8.070	0.005
Low	37	8.14 (6.53-9.74)			12.87 (10.47-15.26)		
Normal	145	9.94 (8.44-11.44)			19.25 (16.75-21.74)		
Albumin			1.185	0.276		2.645	0.104
Low	6	6.50 (3.04-9.96)			10.50 (7.08-13.92)		
Normal	176	9.68 (8.40-10.95)			18.21 (16.07-20.34)		
CEA			0.128	0.721		0.830	0.362
High	39	9.51 (6.47-12.56)			19.46 (13.76-25.16)		
Normal	143	9.59 (8.23-10.94)			17.54 (15.39-19.69)		
NSE			2.344	0.126		2.129	0.145
High	86	8.62 (7.27-9.96)			16.45 (13.78-19.13)		
Normal	96	10.43 (8.41-12.45)			19.29 (16.17-22.41)		
TNM stage			24.199	< 0.001		12.132	0.016
Ⅱa	4	24.25 (8.92-57.96)			25.5 (11.45-60.04)		
Ⅱb	3	35.67 (5.38-66.95)			46.00 (11.66-80.34)		
Ⅲa	42	11.41 (8.53-14.28)			19.98 (16.25-23.7)		
Ⅲb	54	8.76 (7.17-10.35)			19.65 (15.28-24.01)		
Ⅳ	79	7.39 (6.58-8.20)			14.42 (12.03-16.80)		
Pathological type			0.002	0.962		0.417	0.518
Simple	166	9.48 (8.28-10.69)			17.67 (15.54-19.80)		
Mixed	16	10.50 (3.74-17.26)			20.87 (12.35-29.40)		
Liver metastasis			13.32	< 0.001		17.387	< 0.001
Yes	21	5.67 (4.39-6.95)			10.05 (7.73-12.37)		
No	161	10.08 (8.70-11.46)			18.98 (16.70-21.26)		
Brain metastasis			4.082	0.043		0.909	0.340
Yes	9	6.11 (4.87-7.35)			13.89 (8.96-18.81)		
No	173	9.75 (8.45-11.05)			18.16 (15.99-20.33)		
Adrenal metastasis			2.948	0.086		3.230	0.072
Yes	11	6.64 (5.00-8.27)			11.82 (8.09-15.55)		
No	171	9.76 (8.45-11.08)			18.35 (16.16-20.53)		
Bone metastasis			1.064	0.302		0.843	0.359
Yes	28	8.11 (6.89-9.53)			15.25 (12.05-18.45)		
No	154	9.84 (8.39-11.28)			18.44 (16.06-20.82)		
Pulmonary metastasis			3.716	0.054		5.750	0.016
Yes	19	7.21 (6.01-8.41)			12.16 (8.56-15.75)		
No	163	9.85 (8.47-11.22)			18.63 (16.36-20.89)		
Pleural metastasis			0.383	0.536		0.171	0.680
Yes	16	8.25 (6.06-10.44)			20.00 (11.33-28.67)		
No	166	9.70 (8.35-11.05)			17.75 (15.63-19.88)		
Number of metastatic organs			3.240	0.072		2.390	0.122
Single	41	7.95 (6.71-9.19)			16.02 (12.09-19.96)		
Multiple	29	6.48 (5.42-7.54)			11.93 (9.24-14.62)		
Chemotherapy regimen			1.769	0.184		0.824	0.364
Two drug group	144	9.20 (7.88-10.53)			17.52 (15.27-19.77)		
Three drug group	38	10.97 (7.78-14.17)			19.58 (14.41-24.75)		
Chemotherapy cycle number			40.651	< 0.001		34.566	< 0.001
≤4	78	6.26 (5.14-7.37)			11.94 (10.05-13.82)		
> 4	104	12.06 (10.18-13.94)			22.46 (19.38-25.55)		
Effect of chemotherapy			77.185	< 0.001		9.191	0.010
CR+PR	132	10.14 (8.73-11.55)			19.11 (16.55-21.68)		
SD	40	9.43 (6.38-12.47)			16.13 (12.28-19.97)		
PD	10	2.70 (1.87-3.53)			9.9 (5.47-14.33)		
Thoracic radiotherapy			39.514	< 0.001		31.095	< 0.001
Yes	78	13.40 (10.90-15.89)			24.42 (20.61-28.24)		
No	104	6.70 (5.96-7.45)			13.10 (11.35-14.84)		
SCLC: small cell lung cancer; PFS: progression-free survival; OS: overall survival; ECOG PS: Eastern Cooperative Oncology Group performance status; CEA: carcino-embryonic antigen; NSE: neuron specific enolase; CR: complete response; PR: partial response; SD: stable disease; PD: progressive disease.							

**2:** 两药组中卡铂组与顺铂组PFS及OS的比较 Comparison of PFS and OS between the carboplatin group and the cisplatin group in the two drug group

	*n*	PFS (95%Cl)(month)	*χ*^2^	*P*	OS (95%Cl)(month)	*χ*^2^	*P*
Carboplatin	60	8.57 (6.53-10.60)	1.253	0.263	17.03 (13.35-20.72)	0.315	0.574
Cisplatin	84	9.66 (7.90-11.41)			17.87 (15.04-20.70)		

**3:** 胸腔放疗介入时间及放化疗方式对PFS及OS的影响 The effect of thoracic radiotherapy intervention time and mode of radiotherapy and chemotherapy on PFS and OS

	*n*	PFS (95%Cl)(month)	*χ*^2^	*P*	OS (95%Cl)(month)	*χ*^2^	*P*
Intervention time		0.911	0.340		1.525	0.217
Early	55	13.06 (9.99-16.11)			22.75 (18.36-27.13)		
Late	23	14.22 (9.89-18.55)			28.44 (20.95-35.92）		
Mode			3.474	0.062		1.134	0.287
Synchronous	14	11.21 (4.13-18.30)			20.29 (11.85-28.72)		
Sequential	64	13.88 (11.24-16.51)			25.33 (21.06-29.60)		

对发病时非脑转移患者的进一步分析显示，接受PCI组与未接受PCI组其PFS及OS存在统计学差异（表 4）。对非脑转移患者是否行PCI与之后发生脑转移情况进行双变量相关分析显示非脑转移患者是否行PCI与之后是否出现脑转移间的*Pearson*相关系数为-0.163（*P*=0.032），提示存在负相关。

**4:** 非脑转移患者是否行PCI对PFS及OS的影响 The effect of whether or not receiving PCI in the non-brain metastasis group on PFS and OS

	*n*	PFS (95%Cl)(month)	*χ*^2^	*P*	OS (95%Cl)(month)	*χ*^2^	*P*
PCI	23	17.09 (10.97-23.21)	12.518	< 0.001	30.35 (23.62-37.08)	11.567	0.001
Non-PCI	150	8.63 (7.55-9.71)			16.29 (14.16-18.43)		
PCI: prophylactic cranial irradiation.							

#### 2.2.3 多因素分析

采用*Cox*比例风险模型对单因素分析中有意义的变量进行多因素分析，其中影响PFS的因素为一线化疗周期数、初始化疗疗效、胸腔放疗与否（*P* < 0.05）（表 5）。影响OS的因素为血红蛋白水平、肝转移与否、肺内转移与否、一线化疗周期数、胸腔放疗与否（表 6）。

**5:** SCLC患者PFS影响因素的多因素分析 Multivariate analysis of PFS in SCLC patients

Factor	B	SE	Wald *χ*^2^	*P*	Exp(B)
Chemotherapy cycle number	-0.551	0.177	9.748	0.002	0.576
Effect of chemotherapy			33.278	< 0.001	
SD	-2.172	0.387	31.538	< 0.001	0.114
PD	-1.846	0.419	19.392	< 0.001	0.158
Thoracic radiotherapy	-0.535	0.189	8.005	0.005	0.585

**6:** SCLC患者OS影响因素的多因素分析 Multivariate analysis of OS in SCLC patients

Factor	B	SE	Wald *χ*^2^	*P*	Exp(B)
Hemoglobin	0.498	0.197	6.377	0.012	1.646
Liver metastasis	0.605	0.274	4.885	0.027	1.831
Pulmonary metastasis	0.646	0.282	5.248	0.022	1.908
Number of chemotherapy cycle	-0.605	0.190	10.128	0.001	0.546
Thoracic radiotherapy	-0.472	0.197	5.743	0.017	0.624

## 3 讨论

SCLC对初始放化疗敏感，但容易出现复发及进展，生存期短。是否出现复发/进展后就直接意味着生存期缩短尚缺少相关文献报道。本研究对两者的双变量相关分析显示，PFS与OS间存在显著正相关。表明寻找SCLC患者复发/进展的因素，延长患者PFS，对提高生存率有重要意义。

SCLC的发病大部分与吸烟有关。ECOG PS评分与SCLC患者的生存时间相关。本研究均未显示吸烟情况、ECOG PS评分、年龄、性别与PFS及OS之间的统计学相关性。

肿瘤TNM分期系统有助于帮助制定治疗计划、判断患者预后以及评估治疗效果，起初多用于非小细胞癌。SCLC主要采用美国退伍军人肺癌研究组制定的分期方法为局限期及广泛期（limited-stage disease/extensive-stage disease, LD/ED），而近年来，随着外科手术治疗地位在SCLC中的再评估，认为当评估可手术治疗的SCLC时，TNM分期比LD/ED分期更有用^[2]^。TNM分期对SCLC PFS是否存在影响，尚无文献报道。本研究中单因素分析表明不同的TNM分期其PFS及OS存在统计学差异，总体趋势上PFS及OS Ⅳ期 < Ⅲ期 < Ⅱ期，Ⅲb期 < Ⅲa期，而IIb期 > Ⅱa期，不排除与例数少有关，需要进一步的临床论证。但在多因素分析中，TNM分期非影响PFS及OS的独立因素。

肝脏是人体最大实质性脏器，一旦出现肝转移，疾病进展速度快，可能与肝脏本身解剖特征和双重血供的特点有关。一项旨在研究广泛期SCLC患者生存预测因子的回顾性分析^[3]^中，多因素*Cox*回归分析显示肝转移、全部化疗周期的次数是生存率的独立预后因素。肝转移患者风险比无肝转移患者高出2.52倍（*P* < 0.001）。本研究182例SCLC患者中21例合并肝转移，合并肝转移者PFS（5.67个月）与未发生肝转移患者（10.08个月）在单因素分析中存在差异（*P* < 0.001）。单因素分析中OS在肝转移组与非肝转移组同样存在差异（分别为10.08个月、18.98个月；*P* < 0.001）。而多因素分析中肝转移与否非影响PFS的独立因素，但为影响OS的独立因素。肺内转移与PFS不存在统计学差异，但为影响OS的因素。单因素分析中脑转移与否其PFS存在差异，其OS不存在差异，但多因素分析表明脑转移非影响PFS的独立因素。其他器官转移（肾上腺、骨、胸膜）以及转移器官数目（单个或多个器官转移）与PFS及OS之间无统计学差异（*P* > 0.05）。

90%SCLC的病理类型单一，其余为混合型（含有腺癌、鳞癌或大细胞癌等成分），单一病理类型的SCLC对放化疗很敏感而混合型敏感性差，小细胞癌杀灭后剩余的非小细胞成分为复发的根源之一。对1997年-2009年间法国格勒诺布尔大学医院收治的300例SCLC患者的特征以及不同治疗阶段的治疗反应的分析显示，一线化疗后，难治组与敏感组相比，其广泛期多（76.6% *vs* 34.3%, *P*=0.003）、PS评分差（PS 0-1: 48.4% *vs* 67.8%, *P*=0.008）、内分泌副癌综合征多（18.7% *vs* 8.4%, *P*=0.03）以及混合病理成分多（17.2% *vs* 4.9%, *P*=0.004）^[4]^。本研究中，166例（91.2%）患者为单纯型SCLC，16例为复合型SCLC，与SCLC PFS及OS之间无统计学差异（*P* > 0.05）。

血清肿瘤标志物在部分恶性疾病处理中起着重要作用，包括早期诊断、决定预后、预测对特定治疗敏感或耐药。NSE是SCLC的敏感指标（本研究中86例患者出现NSE不同程度的升高），CEA在部分SCLC患者中亦可出现升高（本研究中有39例患者升高）。本研究结果显示，发病时NSE、CEA是否增高与PFS及OS无统计学差异（*P* > 0.05）。

低钠血症可在约15%的SCLC患者中发生。低钠血症患者其中位生存期显著缩短，低钠血症是SCLC（ED及LD）患者死亡率的独立危险因素^[5]^。低血红蛋白与预后差有关^[6]^，其导致的乏氧细胞对化放疗不敏感、耐受性差可能是导致生存期短的主要原因。LDH与化疗疗效、生存期密切相关，并与肿瘤负荷相关。多因素分析^[7]^证实疾病进展、PS评分以及血LDH水平为影响生存期的独立预后因素。有肝转移和/或PS评分2分和/或白蛋白低者，不管无化疗缓解期如何，均有相似的预后差^[8]^。本研究拟分析以上指标（是否低钠血症、LDH水平增高与否、是否低蛋白血症、是否低血红蛋白血症）与PFS及OS的相关性。结果显示低钠血症、LDH增高与否、是否低蛋白血症与PFS及OS无统计学差异（*P* > 0.05）。血红蛋白下降者其PFS短，但未达统计学意义。血红蛋白下降为影响OS的独立因素。

化疗是SCLC的主要治疗方法。本研究中一线化疗周期数为影响SCLC PFS及OS的独立因素，大于4个周期的化疗PFS及OS时间延长。单因素分析及多因素分析显示初始化疗疗效是影响PFS的独立因素。初始化疗疗效在单因素分析中为OS的影响因素，但多因素分析显示非独立因素。EP/EC为SCLC的主要一线化疗方案，本研究两药组中卡铂组与顺铂组的生存情况，结果显示两者对PFS及OS无统计学差异（*P* > 0.05）。三药组的PFS及OS长于两药组，但未达统计学意义。

早期放疗将减轻肿瘤克隆原性细胞的再增殖效应，在发生远处转移前消灭耐药癌细胞，从而提高局控率和减少远处转移率^[9]^。本研究182例患者中，78例接受了胸腔放疗，放疗者与非放疗者PFS及OS分别为（13.40个月*vs* 6.7个月；24.42个月*vs* 13.10个月），存在统计学差异（*P* < 0.001）。而胸腔放疗介入的时间及放化疗方式（序贯、同步）与PFS及OS之间无统计学差异（*P* > 0.05）。多因素分析显示胸腔放疗是影响PFS及OS的独立因素。

脑是SCLC最常见的转移部位之一^[10]^，在确诊SCLC时至少18%的患者已出现脑转移，随着疾病的进展，2年脑转移的发生率增加至80%。由于血脑脊液屏障的存在，使脑组织中抗癌药物的浓度明显低于脑外组织，使脑成为癌细胞逃避化疗药物杀灭的避难所，也成为SCLC治疗失败的主要原因之一。Manapov^[11]^总结了4例以脑转移症状为首发表现的广泛期SCLC的患者资料。所有患者均在起始的同步颅脑化放疗后联合化疗及胸部化放疗并取得成功，但在初始治疗后很快出现（即使接受二线治疗）一系列中枢神经系统转移症状。中枢神经系统症状成为疾病的主导，尽管在最终随访阶段所有4例患者的原发肿瘤仍保持完全缓解。预防性颅脑照射在局限期SCLC患者及对初始治疗反应相当好的广泛期SCLC患者中的应用被认为是近期SCLC处理中的最主要进展。本研究结果显示，非脑转移患者是否行PCI对PFS及OS存在差异（17.09个月*vs* 8.63个月，*P* < 0.001；30.35个月*vs* 16.29个月，*P*=0.001）；是否行PCI与之后是否发生脑转移存在显著负相关。

综上所述，初治非手术SCLC的复发/进展时间与发病时肿瘤状态（TNM分期、是否肝转移、是否脑转移）存在相关性，而治疗因素（一线化疗周期数 > 4次、行胸腔放疗、初始化疗效果佳、非脑转移患者行PCI）可延长复发/进展时间，且一线化疗周期数、初始化疗疗效以及胸腔放疗为影响复发/进展时间的独立因素。复发/进展时间与总生存时间存在显著正相关。提示着我们，对一些存在高危因素患者（如TNM分期晚、存在肝转移、存在脑转移），若身体状况许可，可能通过增加一线化疗周期数、联合胸腔放疗等延长复发/进展时间。对初始治疗效果好的患者可行PCI治疗以延长复发/进展时间，减少脑转移概率。对于SCLC患者，应根据发病时情况，进行更为个体化的治疗，从而提高肿瘤的PFS，进一步延长OS。
